# Arterial Stiffness Is Associated with Peripheral Sensory Neuropathy in Diabetes Patients in Ghana

**DOI:** 10.1155/2018/2320737

**Published:** 2018-01-31

**Authors:** Kwame Yeboah, Jennifer A. Agyekum, Richard N. A. Owusu Mensah, Patrick K. Affrim, Linda Adu-Gyamfi, Rita O. Doughan, Afua B. Adjei

**Affiliations:** ^1^Department of Physiology, School of Biomedical & Allied Health Sciences, University of Ghana, Accra, Ghana; ^2^Department of Medical Laboratory Sciences, School of Biomedical & Allied Health Sciences, University of Ghana, Accra, Ghana; ^3^Department of Chemical Pathology, School of Biomedical & Allied Health Sciences, University of Ghana, Accra, Ghana; ^4^Department of Medical Biochemistry, School of Biomedical & Allied Health Sciences, University of Ghana, Accra, Ghana

## Abstract

**Objective:**

Peripheral sensory neuropathy (PSN) is among microvascular complications of diabetes that make patients prone to ulceration and amputation. Arterial stiffness is a predictor of cardiovascular diseases and microvascular complications associated with diabetes. We investigated the association between PSN and arterial stiffness, measured as aortic pulse wave velocity (PWVao) and cardio-ankle vascular index (CAVI).

**Method:**

In a case-control design, arterial stiffness was measured in 240 diabetes patients and 110 nondiabetic control. Large-fibre nerve function was assessed by vibration perception threshold (VPT) using a neurothesiometer. PSN was defined as the VPT > 97.5th percentile from age- and gender-adjusted models in nondiabetic controls.

**Results:**

The overall prevalence of PSN was 16.6% in the entire study participants. Compared to non-PSN participants, PSN patients had higher levels of PWVao (9.5 ± 1.7 versus 8.7 ± 1.2 m/s, *p* = 0.016) and CAVI (8.4 ± 1.3 versus 7.6 ± 1.1, *p* = 0.001). In multiple regression models, VPT was associated with PWVao (*β* = 0.14, *p* = 0.025) and CAVI (*β* = 0.12, *p* = 0.04). PSN patients had increased odds of CAVI (OR = 1.51 (1.02–2.4), *p* = 0.043), but not PWVao (OR = 1.25 (0.91–1.71), *p* = 0.173).

**Conclusion:**

PWVao and CAVI were associated with VPT and PSN in diabetes patients in Ghana. Patients having PSN have increased odds of CAVI, independent of other conventional risk factors.

## 1. Introduction

Diabetes is now common in sub-Saharan African population and associated with macrovascular and microvascular complications [1, 2]. Cardiovascular diseases are among the major causes of death in diabetes patients; however, studies have shown that microvascular disease is also associated with excess mortality [3]. Coexistence of cardiovascular and microvascular diseases in diabetes patients is associated with higher mortality [4, 5]. Peripheral sensory neuropathy (PSN) in the lower limbs is a classic example of microvascular complication of diabetes, with a high risk for nontraumatic limb amputation, which occurs in 1-2% of diabetic patients and necessitates extreme cost [6].

Diabetes patients in Ghana have abnormalities in arterial function that can be measured using aortic pulse wave velocity (PWVao) or cardio-ankle vascular index (CAVI) [7, 8]. Both PWVao and CAVI measure arterial stiffness, with CAVI assessing the stiffness in long range of arterial segment (from the aortic root to the ankle arteries), as opposed to PWVao, which assesses the stiffness in the descending aorta, from the aortic root to the bifurcation. Arterial stiffness predicts cardiovascular and all-cause mortality in diabetes patients [9, 10]. The association between arterial stiffness and PSN in diabetes patients in sub-Saharan Africa is unknown. We, therefore, investigated the levels of various indices of arterial stiffness in PSN patients and the association between PSN and arterial stiffness. We hypothesize that, compared to non-PSN individuals, PSN patients have higher levels of arterial stiffness.

## 2. Materials and Methods

### 2.1. Study Design and Subjects

This study was a case control design, conducted within the period of December 2012 to June 2013, at the Korle-Bu Teaching Hospital in Accra, which is a 1500-bed tertiary hospital and serves as the main referral hospital in Ghana. The study population were selected from two sources: (1) diabetes patients, selected systematically as every 3rd consecutive patient visiting the diabetes clinic and consented to take part in the study, and (2) nondiabetic individuals, invited from the surrounding communities and conveniently recruited into the study. Vibration perception threshold and arterial stiffness were assessed in all the study participants. Individuals with nontraumatic limb amputation and those unable to comprehend and comply with the protocol requirements (psychological and/or cognitive disorders, failure to cooperate, and failure to sign the informed consent document) were excluded from the study. In all, 350 subjects, comprising 240 diabetes patients and 110 nondiabetic individuals, were screened for PSN. The study was approved by the University of Ghana Medical School Ethical and Protocol Review Committee (protocol ID number: MS-Et/M.2 – P.4.10/2012-2013) and all participants gave written informed consent after the procedures involved in the study were thoroughly explained to them, following the general recommendations of the Declaration of Helsinki.

#### 2.1.1. Anthropometric Measurements

Using standard protocols [11], body weight was determined twice using a homologated electronic scale (Seca 770, Hamburg, Germany) following due calibration (precision ± 0.1 kg), with the patient wearing light clothing and shoes removed. Height was also measured with a portable system (Seca 222, Hamburg, Germany) with the patient shoeless in the upright position. Body mass index (BMI) was calculated as weight (kg) divided by height squared (m^2^). Waist circumference was measured with a nonelastic tape measure at the upper border of the iliac crest, parallel to the floor without compressing the skin. Body composition monitor (BF508, Omron Healthcare Inc., Vernon Hills, IL, USA) was used to assess body fat percentage.

#### 2.1.2. Biochemical Analysis

Blood samples were drawn in the morning, after 8–12 hours of overnight fasting, into plain vacuum tubes to measure plasma lipids and fluoride oxalate tubes for glucose levels. FPG, 2h-PPG, total cholesterol, high-density lipoprotein cholesterol (HDL), and triglyceride levels were analysed by colorimetric enzymatic assays using BS 120 chemical autoanalyser (Mindray, China) and commercial reagents (Randox Laboratory Reagents, UK). Low-density lipoprotein cholesterol levels were calculated using Friedewald's formula [12]. All analyses were performed at the Diabetes Research and Chronic Disease Reference Laboratory.

### 2.2. Arterial Stiffness Assessment

Arteriograph (TensioMed Kft., Hungary) was used to measure aortic pressure indices, aortic PWV (PWVao), and aortic augmentation index (AIx), with the subject lying supine for at least 10 minutes in a temperature-controlled room (22 ± 2°C). The Arteriograph cuff was applied on the right arm over the brachial artery to detect arterial wall oscillations in the upper arm using the “stop-flow” principle previously described [13]. To determine PWVao, the Arteriograph uses the physiological behaviour of the wave reflection; the ejected direct (first systolic) pulse wave is reflected back mostly from the aortic bifurcation. The device measures the time interval between the peaks of the direct (first) and reflected (late) systolic wave (return time). For both the invasive and noninvasive PWVao calculation, the distance from the sternal notch (jugulum) to the upper edge of the pubic bone (symphysis) is used because this provides the nearest value of the true aortic path length [14, 15]. To avoid overestimation of aortic path length by body surface contours, a specialised body calliper was used to measure the jugulum–symphysis distance.

The aortic PWV was calculated by using the following formula:

(1) Aortic PWV /sm=2jugulum−symphysis distancereturn time.

Augmentation index (AIx) is computed using the algorithm below:

(2) $$\begin{array}{c} AIx=\frac{P_{2}-P_{1}}{PP}\times 100, \end{array}$$

where *P*_1_ is the amplitude of the first (direct) wave, *P*_2_ is the amplitude of the late (reflected) systolic wave, and PP is the pulse pressure.

CAVI and heart-ankle (ha) PWV were measured using the Vasera 1500N (Fukuda-Denshi, Japan) with the participant resting supine for at least 10 minutes before the measurement. Electrocardiogram electrodes were placed on both wrists, a microphone for detecting heart sounds placed on the sternum, and cuffs were wrapped around the upper arms and the ankles. CAVI values were computed automatically. Briefly, CAVI corresponds to the stiffness parameter *β*, calculated from values of ha-PWV and BP as follows:

(3) $$\begin{array}{c} \beta =\left(\frac{2\rho }{\Delta P}\right)\cdot \left[\ln\left(\frac{P_{s}}{P_{d}}\right)\right]{PWV}^{2}, \end{array}$$

where *ρ* indicates blood density; Δ*P*, pulse pressure; ln, natural log; *P*_s_, systolic BP; and *P*_d_, diastolic BP [16, 17]. CAVI and Arteriograph measurements were performed in a random order using a digital algorithm.

### 2.3. Neurothesiometry

Neurothesiometry was performed using hand-held neurothesiometer (Horwell Neurothesiometer, Scientific Laboratory Supplies Ltd., Nottingham, UK) to the read vibration perception threshold (VPT) from the apex of the big toe of both legs, with the subject in a supine position, feet elevated with pillow support, and eyes closed. The neurothesiometer is a validated battery-operated diagnostic instrument that assesses sensitivity thresholds to vibration corresponding to a particular voltage at various sites on the body surface. On the basis of the method of limits, participants were asked to indicate when they first perceived vibration sensation after the stimulus was applied to the distal pulp of the toe. The intensity of the stimulus was gradually increased at a rate of 0.5 V/s from null to a voltage at which vibration was first detected. VPT was performed on each participant about 3–5 times and, at least, three VPTs that differed ≤5 V were averaged and used for analysis. A null stimulus test was randomly performed to ensure that the participant understands and adheres to the test procedure. Participants who failed to provide 3 consistent values of VPT within 5 V after seven measurements were excluded from the analysis, considered as having conflicting VPTs.

### 2.4. Statistical Analysis

Continuous data were analysed with the Shapiro-Wilk test to determine their distribution, and skewed variables were logarithmically transformed before analysis. The cut-offs for PNS were computed as the VPT ≥ 97.5th percentile from age- and gender-adjusted regression models in the nondiabetic controls. From the regression models, VPT > 24 V in males and >21 V in females were used to define PNS. Variables with normal distribution were presented as mean ± standard deviation and analysed using Student's *t*-test or analysis of variance, as deemed appropriate. Variables with nonnormal distribution were presented as median and interquartile range and analysed using Mann–Whitney *U* test or Kruskal-Wallis test, as deemed appropriate. Categorical data were analysed by the *χ*^2^ test. Zero-order and partial Pearson's correlation tests were used to test the association between VPT versus arterial stiffness indices and patient characteristics. Multivariable linear and logistic regression models were performed to analyse the association between VPT/PSN and indices of arterial stiffness.

## 3. Results

The prevalence of PSN in our study population was 16.6%, higher in diabetes patients. Compared to nondiabetic individuals, diabetes patients have higher heart rate, pulse pressure, total and LDL cholesterol, PWVao, aortic systolic BP, aortic pulse BP, CAVI, ha-PWV, and VPT. Diabetes patients had lower HDL cholesterol and AIx (Table 1). Compared to non-PSN participants, PSN patients were taller and had higher duration of diabetes, higher systolic and pulse BP, and higher heart rate (Table 2). VPT correlated positively with age, duration of diabetes, waist-hip ratio, and fasting plasma glucose and negatively with body fat (Table 3).

**Figure 1:**
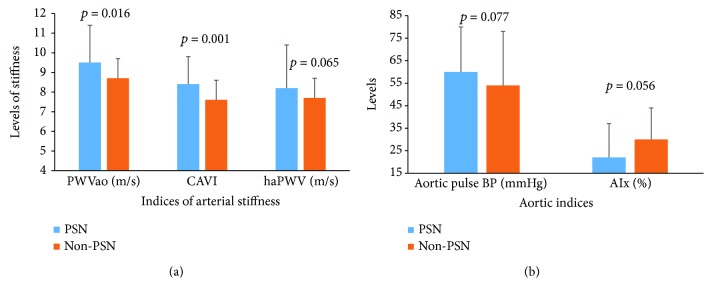
(a) Comparison of arterial stiffness indices by PSN status. Bars represent means and error bars represent standard deviations. (b) Comparison of aortic indices by PSN status. Bars represent means and error bars represent standard deviations.

**Figure 2:**
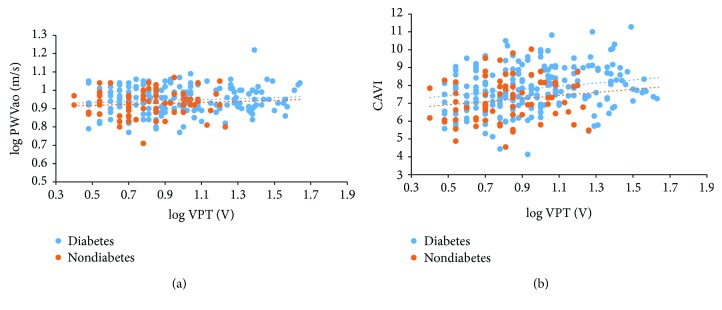
(a) Correlation between log VPT and log PWVao in diabetes and nondiabetes participants. Data analysed by Pearson's correlation test. In nondiabetes participants, log VPT do not correlate with log PWVao in zero-order (*r* = 0.13, *p* = 0.313) and age and gender partial (*r* = −0.1, *p* = 0.444) correlations. In diabetes patients, log VPT correlated with log PWVao in zero-order (*r* = 0.14, *p* = 0.013) and age and gender partial (*r* = 0.16, *p* = 0.004) correlations. (b) Correlation between log VPT and CAVI in diabetes and nondiabetes participants. Data analysed by Pearson's correlation test. In nondiabetes participants, log VPT do not correlate with CAVI in zero-order (*r* = 0.16, *p* = 0.186) and age and gender partial (*r* = −0.05, *p* = 0.69) correlations. In diabetes patients, log VPT correlated with log CAVI in zero-order (*r* = 0.21, *p* = 0.002) and nonsignificant in age and gender partial (*r* = 0.12, *p* = 0.067) correlations.

With respect to the indices of arterial stiffness, compared to non-PSN participants, PSN patients had higher means of PWVao, (Figures 1(a) and 1(b)). In the entire participants, log VPT was associated with log PWVao in zero-order (*r* = 0.14, *p* = 0.026) and age and gender partial (*r* = 0.12, *p* = 0.048) correlations. Similarly, log VPT was associated with CAVI in zero-order (*r* = 0.24, *p* < 0.001) and age and gender partial (*r* = 0.12, *p* = 0.04) correlations. When the participants were grouped by diabetes status, log VPT correlated with log PWVao and CAVI in diabetes patients, but not significant in nondiabetes participants (Figures 2(a) and 2(b)).

In multivariable linear regression analysis, with confounders forced into the model, VPT was associated with PWVao, CAVI, and haPWV. Other factors like age, body height, and diabetes status were also associated with VPT (Table 4). In logistic regression models, PSN patients have increased odds of change in PWVao and CAVI in unadjusted models. In adjusted models, PSN patients have increased odds of change in CAVI and decreased odds of change in AIx. The contributions of other variables in the models were indicated in the footnote (Table 5).

## 4. Discussion

The findings of this study showed that compared to participants without PSN, PSN patients had higher levels of PWVao and CAVI. Also, VPT increases significantly with PWVao, CAVIs, and ha-PWV in multiple regression models, after adjusting for confounders. However, only CAVI was increased in the multivariable logistic regression model. These findings are similar to other studies that reported association between neuropathy and arterial stiffness; though most of these studies utilized subjective neurological examination for diagnosis of neuropathy [18, 19]. A recent cross-sectional study reported that carotid-femoral PWV, which is the gold standard for arterial stiffness, was associated with severity of neuropathy assessed using the neuropathy symptom score instrument [20]. Yokoyama et al. [18] reported that ba-PWV, an index similar to ha-PWV, increases together with levels of neuropathy assessed by neuropathic symptoms and tuning fork vibration. In Korean diabetes patients, Kim et al. [19] reported that neuropathy, assessed as neuropathic symptoms and current perception threshold, was associated with CAVI. Likewise, the Rio de Janeiro Type 2 Diabetes Cohort Study, which utilized the longitudinal design, reported that patients with high carotid-femoral PWV (>10 m/s) had twice the risk of being clinically diagnosed with peripheral neuropathy during a median follow-up of 6.2 years [21]. As indicated by adjusted *R*^2^ values in multiple linear regression models, the indices of arterial stiffness might be able to explain up to 25% of the variations in the VPT in our study participants. Also, the association between CAVI and PSN in multivariable logistic regression implies that stiffness in the long arterial path might be a significant determinant of PSN.

The link between arterial stiffness and neuropathy can be explained by the unique anatomy of vascular supply to peripheral nerves. Peripheral nerves, in contrast to the central nervous system, are supplied by the upper branches of arteries supplying musculature of the limbs. Hence, it could be expected that stiffness in the peripheral arteries can affect the peripheral nerves as well. Also, as reported by Smith et al. [22], vascular supply to peripheral nerves are sparse and lacks autoregulation, making the nerves vulnerable to ischemia as a result of stiffness in elastic arteries. In addition, Edmonds et al. [23] and Young et al. [24] observed higher levels of arterial medial calcification in diabetes patients with neuropathic symptoms when compared to diabetes patients without neuropathy. This means that diabetes causes arterial medial calcification, resulting in increased arterial stiffness, which might also lead to neuropathy [25].

The findings of this study showed that the prevalence of neuropathy was 16.4% higher in diabetes patients. VPT assess the function of large-fibre nerves, and hence, high VPT may be an indication of large-fibre nerve damage [4]. This method had been found to predict foot ulceration in high-risk subjects [26]. The prevalence of PSN in diabetes patients in Sweden was 34% [27] and 25.5% in Singapore [28]. The low prevalence of PNS in our study might be that diabetes-related neuropathy is presented differently in diabetes patients in Ghana. Also, there might be a possibility that the subjects did not understand the protocol well and/or gave a false feedback about perceived vibration sensation [29]. The utility and validity of neurothesiometer to detect PSN in sub-Saharan Africans need to be determined in a longitudinal study design. The major limitation of neurothesiometry in screening for PSN is the subject's dependency, and this can be overcome by using the objective form of assessment such as the nerve conduction study [30].

The findings of this study also show that VPT correlated with age, duration of diabetes, body fat, WHR, and FPG. Also, in the multivariable analyses, age, gender, and body height emerged as significant predictors of PSN and VPT. This is similar to the findings of Bergenheim et al. [31], who reported an association of diabetic neuropathy with age, body height, and duration of diabetes. Tesfaye et al. [32] in the EURO-Diab group reported that blood glucose control, duration of diabetes, and hyperlipidaemia were significant risk factors for the development of neuropathy in type 1 diabetes patients. Contrary to the findings of Tesfaye et al., plasma lipids did not correlate with VPT in this study. Our findings were similar to that of Yagihashi et al. [33], who reported that plasma levels of triglycerides or cholesterol were not associated with diabetic neuropathy.

### 4.1. Limitations of the Study

In this study, PSN was assessed employing quantitative vibration testing in the form of VPT. VPT assess the acuity of somatosensory pathways responsible for transmitting information induced by cutaneous vibratory stimuli, being neuroselective for large and myelinated A*α* and A*β* sensory fibres [34]. Therefore, VPT fails to assess the functional status of small and medium nerves. Another notable caution about VPT is that it is used to assess probable neuropathy and nerve conduction studies are required for confirmation [35]. This study was a cross-sectional design, and hence, we cannot infer causation from our findings, whether arterial stiffness precedes the development of neuropathy or neuropathy causes arterial dysfunction in diabetes patients. Also, the participants of the study were recruited from a tertiary health facility, implying that the findings may differ from diabetes patients from primary healthcare facilities. Therefore, we cannot infer the conclusions to the entire Ghanaian population. It will be interesting for future studies to investigate the association between arterial stiffness and PSN in diabetes patients from other levels of health care facilities, and utilizing the longitudinal study design.

## 5. Conclusion

Our findings indicate that arterial stiffness, measured as PWVao and CAVI, was associated with VPT and PSN in diabetes patients in Ghana. Patients having PSN have increased odds of CAVI, independent of other conventional risk factors.

## Figures and Tables

**Table 1:** General characteristics of study participants by diabetes status.

	All participants (*n* = 350)	T2DM (*n* = 240)	Non-DM (*n* = 110)	*p*
Females, *n* (%)	175 (50)	108 (45)	67 (60.1)	0.15
Hypertension, *n* (%)	179 (52.6)	137 (57.1)	42 (38.2)	
Age (yrs)	54.1 ± 10.2	53.7 ± 10.1	54.6 ± 10.3	0.54
Duration of diabetes (yrs)	10.4 ± 7.8	10.4 ± 7.8		
Weight (kg)	79.5 ± 14.9	79.9 ± 15.5	79 ± 14.3	0.672
Height (cm)	166 ± 8.4	167 ± 8	164 ± 9	0.061
BMI (kg/m^2^)	29.1 ± 5.7	28.9 ± 5.9	29.4 ± 5.5	0.571
Waist circumference (cm)	98 ± 14	99 ± 12	96 ± 15	0.073
Waist-hip ratio	0.91 ± 0.11	0.92 ± 0.07	0.9 ± 0.14	0.382
Systolic BP (mmHg)	139 ± 30	141 ± 26	135 ± 34	0.174
Diastolic BP (mmHg)	83 ± 13	83 ± 13	82 ± 14	0.594
Pulse BP (mmHg)	59 ± 14	59 ± 14	58 ± 13	0.485
Heart rate (bpm)	71 ± 17	75 ± 13	65 ± 19	<0.01
FPG (mmol/l)	6.9 ± 3.2	8.4 ± 2.9	5 ± 2.5	<0.01
2h-PPG (mmol/l)	7.8 ± 1.4		7.8 ± 1.4	
Triglycerides (mmol/l)	1.1 ± 0.5	1.1 ± 0.5	1.2 ± 0.6	0.586
Total cholesterol (mmol/l)	4.7 ± 1.5	5.5 ± 1.4	3.9 ± 1.1	<0.001
HDL cholesterol (mmol/l)	0.9 ± 0.2	0.7 ± 0.2	1.2 ± 0.4	0.025
LDL cholesterol (mmol/l)	3.2 ± 1.4	3.9 ± 1.3	2.7 ± 1.4	<0.001
PWVao (m/s)	8.8 ± 1.6	9 ± 1.5	7.8 ± 1.7	0.006
AIx (%)	29 ± 14.6	27.1 ± 14.7	34 ± 12.9	<0.001
Aortic systolic BP (mmHg)	143 ± 29	137 ± 28	145 ± 29	0.034
Aortic pulse BP (mmHg)	55 ± 24	58 ± 19	63 ± 26	0.082
CAVI	7.6 ± 1.3	7.8 ± 1.1	7.1 ± 1.1	0.004
haPWV (m/s)	7.7 ± 1.7	7.9 ± 1.9	7.5 ± 1.2	0.094
VPT (V)	11.9 ± 6.2	15.1 ± 7.8	7.3 ± 3.8	<0.001
PSN, *n* (%)	58 (16.6)	47 (19.4)	11 (10)	<0.001

**Table 2:** Comparison of clinical features of study participants by PSN status.

	No PSN (*n* = 292)	PSN (*n* = 58)	*p*
Age (yrs)	54.3 ± 10.2	52.6 ± 10.4	0.529
Females, *n* (%)	154 (52.7)	21 (36.2)	0.063
Diabetes, *n* (%)	193 (66.1)	47 (81)	0.011
Hypertension, *n* (%)	117 (50)	35 (60.3)	0.032
Duration of diabetes	7.7 ± 6.6	14.8 ± 6.8	0.001
Previous smoking, *n* (%)	38 (13)	13 (22.4)	0.15
Weight (kg)	79 ± 14.9	84 ± 15	0.215
Height (cm)	165 ± 8	175 ± 7	<0.001
BMI (kg/m^2^)	29.2 ± 5.7	27.6 ± 5.2	0.257
Body fat (%)	35.1 ± 12.6	28 ± 11.5	0.037
Visceral fat (%)	11.2 ± 4.1	12.4 ± 6	0.324
Waist circumference (cm)	98 ± 13	98 ± 12	0.892
Waist-hip ratio	0.91 ± 0.1	0.92 ± 0.05	0.825
Systolic BP (mmHg)	138 ± 30	154 ± 18	0.006
Diastolic BP (mmHg)	82 ± 13	87 ± 15	0.176
Pulse BP (mmHg)	58 ± 14	66 ± 8	0.002
Heart rate (bpm)	70 ± 17	80 ± 7	<0.001
FPG (mmol/l)	6.9 ± 3.3	6.5 ± 1.9	0.387
Triglycerides (mmol/l)	1.1 ± 0.5	1 ± 0.4	0.205
Total cholesterol (mmol/l)	4.7 ± 1.4	4.5 ± 1.8	0.506
HDL cholesterol (mmol/l)	0.7 ± 0.2	0.7 ± 0.3	0.913
LDL cholesterol (mmol/l)	3.3 ± 1.4	3.2 ± 1.5	0.825

**Table 3:** Correlation between VPT and clinical parameters of participants.

	VPT	
	*r*	*p*
Age	0.23	0.002
Duration of diabetes	0.28	0.004
Body mass index	−0.12	0.110
Body fat	−0.18	0.015
Visceral fat	0.07	0.391
Waist circumference	0.07	0.366
Waist-hip ratio	0.17	0.021
Waist-height ratio	−0.05	0.542
Fasting plasma glucose	0.17	0.018
2h-PPG	0.07	0.574
Total cholesterol	0.05	0.510
Triglycerides	−0.01	0.902
HDL cholesterol	0.008	0.293
LDL cholesterol	−0.01	0.865

**Table 4:** Multiple regression models of VPT and indices of arterial stiffness.

	Unstandardized B	Standardised coefficient (*β*)	Adjusted *R*^2^	*p*
PWVao^1^	0.026 ± 0.104	0.136	0.25 ± 0.24	0.025
CAVI^2^	0.023 ± 0.011	0.121	0.26 ± 0.22	0.04
ha-PWV^3^	0.032 ± 0.013	0.141	0.24 ± 0.23	0.019
AIx	−0.004 ± 0.001	−0.1	0.25 ± 0.24	0.098
Aortic systolic BP	0.009 ± 0.001	0.092	0.24 ± 0.23	0.13
Aortic pulse BP	0.002 ± 0.001	0.052	0.25 ± 0.23	0.387

Adjusted for age, gender, height, BMI, waist circumference, diabetes, hypertension, alcohol, and smoking. PWVao: aortic pulse wave velocity; CAVI: cardio-ankle vascular index; ha-PWV: heart-ankle pulse wave velocity; AIx: aortic augmentation index; BP: blood pressure. ^1^Significant variables in the model were age (*β* = 0.32, *p* < 0.001), body height (*β* = 0.45, *p* < 0.001), and diabetes status (*β* = 0.17, *p* = 0.003). ^2^Significant variables in the model were age (*β* = 0.34, *p* < 0.001), body height (*β* = 0.46, *p* < 0.001), and diabetes status (*β* = 0.17, *p* = 0.001). ^3^Significant variables in the model were age (*β* = 0.31, *p* < 0.001), body height (*β* = 0.48, *p* < 0.001), and diabetes status (*β* = 0.19, *p* = 0.001).

**Table 5:** Logistic regression models of PSN and indices of arterial stiffness.

	Unadjusted OR (95% CI)	*p*	Adjusted OR (95 CI)	*p*
PWVao^1^	1.38 (1.06–1.79)	0.017	1.25 (0.91–1.71)	0.173
CAVI^2^	1.78 (1.26–2.52)	0.001	1.51 (1.02–2.4)	0.043
ha-PWV^3^	1.26 (0.93–1.76)	0.137	1.47 (0.98–2.22)	0.066
AIx^4^	0.97 (0.95–1.01)	0.056	0.95 (0.91–0.98)	0.007
Aortic systolic BP^5^	1.01 (1–1.02)	0.11	0.97 (0.93–1)	0.072
Aortic pulse BP^6^	1.01 (0.99–1.03)	0.238	0.99 (0.97–1.02)	0.76

Adjusted for age, gender, height, BMI, waist circumference, diabetes, hypertension, alcohol, and smoking. PWVao: aortic pulse wave velocity; CAVI: cardio-ankle vascular index; ha-PWV: heart-ankle pulse wave velocity; AIx: aortic augmentation index; BP: blood pressure. The other significant variables in the multivariable-adjusted models were as follows: ^1^Age (OR = 1.08 (1.02–1.15), *p* = 0.01), body height (OR = 1.18 (1.09–2.28), *p* < 0.001), and diabetes status (OR = 8.74 (2.01–12.14), *p* = 0.042). ^2^Gender (OR = 3.65 (1.05–12.99), *p* = 0.046) and body height (OR = 1.18 (1.09–2.28), *p* < 0.001). ^3^Age (OR = 1.06 (1.01–1.12), *p* = 0.037), male gender (OR = 5.1 (1.35–14.65), *p* = 0.017), and body height (OR = 1.19 (1.1–2.29), *p* < 0.001). ^4^Age (OR = 1.12 (1.05–1.19), *p* = 0.037), male gender (OR = 3.6 (1.49–13.45), *p* = 0.012), and body height (OR = 1.18 (1.09–2.29), *p* < 0.001). ^5^Age (OR = 1.09 (1.03–1.16), *p* = 0.004), male gender (OR = 3.98 (1.04–12.17), *p* = 0.044), and body height (OR = 1.18 (1.08–2.28), *p* < 0.001). ^6^Age (OR = 1.08 (1.03–1.15), *p* = 0.004), male gender (OR = 4.28 (1.19–13.38), *p* = 0.026), body height (OR = 1.18 (1.09–2.27), *p* < 0.001), and diabetes status (OR = 4.28 (1.19–9.54), *p* = 0.046).

## Abbreviations

PSN: Peripheral sensory neuropathy
VPT: Vibration perception threshold
PWVao: Aortic pulse wave velocity
CAVI: Cardio-ankle vascular index
ha-PWV: Heart-ankle pulse wave velocity
AIx: Augmentation index
BP: Blood pressure.

## Data Access

The dataset supporting the conclusion of this study is available for systematic review and meta-analysis upon request.
